# Complex ecological interactions of *Staphylococcus aureus* in tampons during menstruation

**DOI:** 10.1038/s41598-018-28116-3

**Published:** 2018-07-02

**Authors:** Isaline Jacquemond, Anaëlle Muggeo, Gery Lamblin, Anne Tristan, Yves Gillet, Pierre Adrien Bolze, Michèle Bes, Claude Alexandre Gustave, Jean-Philippe Rasigade, François Golfier, Tristan Ferry, Audrey Dubost, Danis Abrouk, Samuel Barreto, Claire Prigent-Combaret, Jean Thioulouse, Gérard Lina, Daniel Muller

**Affiliations:** 1 Université de Lyon, Université Claude Bernard Lyon 1, CNRS, INRA, VetAgro Sup, UMR Ecologie Microbienne, 43 bd du 11 Novembre, F-69622 Villeurbanne, France; 2 CIRI, Centre International de Recherche en Infectiologie, Inserm U1111, Université Lyon 1, Ecole Normale Supérieure de Lyon, CNRS UMR 5308 Lyon, France; 3 Department of Gynecology, Hospices Civils de Lyon, Hôpital Femme Mère Enfant, Bron, France; 4 Centre National de Référence des Staphylocoques, Institut des Agent infectieux, Hôpital de la Croix Rousse, Hospices Civils de Lyon, Lyon, France; 5 Department of Pediatric Emergency, Hospices Civils de Lyon, Hôpital Femme Mère Enfant, Bron, France; 6 Department of Gynecological Surgery and Oncology, Obstetrics, Hospices Civils de Lyon, Centre Hospitalier Lyon Sud, Pierre Bénite, France; 7 Service des maladies infectieuses et tropicales, Hôpital de la Croix Rousse, Hospices Civils de Lyon, Lyon, France; 8 Université Lyon 1, CNRS, UMR5558, Laboratoire de Biométrie et Biologie Evolutive, Villeurbanne, France

**Keywords:** Microbiome, Translational research

## Abstract

Menstrual toxic shock syndrome (mTSS) is a severe disease that occurs in healthy women vaginally colonized by *Staphylococcus aureus* producing toxic shock toxin 1 and who use tampons. The aim of the present study was to determine the impact of the composition of vaginal microbial communities on tampon colonisation by *S. aureus* during menses. We analysed the microbiota in menstrual fluids extracted from tampons from 108 healthy women and 7 mTSS cases. Using culture, *S. aureus* was detected in menstrual fluids of 40% of healthy volunteers and 100% of mTSS patients. Between class analysis of culturomic and 16S rRNA gene metabarcoding data indicated that the composition of the tampons’ microbiota differs according to the presence or absence of *S. aureus* and identify discriminating genera. However, the bacterial communities of tampon fluid positive for *S. aureus* did not cluster together. No difference in tampon microbiome richness, diversity, and ecological distance was observed between tampon vaginal fluids with or without *S. aureus*, and between healthy donors carrying *S. aureus* and mTSS patients. Our results show that the vagina is a major niche of*. S. aureus* in tampon users and the composition of the tampon microbiota control its virulence though more complex interactions than simple inhibition by lactic acid-producing bacterial species.

## Introduction

The continuous interactions between an individual host and its microbial community (or microbiota) impact the development of the holobiont^1–3^. The host commensal microbiota is clearly important to host health. In healthy individuals, *Bacteroidetes*, *Proteobacteria*, and *Firmicutes*, such as *Lactobacillales*, are the major phyla among human microbiota^4,5^. These commensals develop complex networks with other microbes and the host, constituting the first line of defence against pathogen invasion by inhibiting colonization^4,6,7^. The contribution of human indigenous microbiota to the control of disease severity and the modulation of pathogen virulence is an emerging issue in the domain of clinical infectiology^8,9^.

*Staphylococcus aureus*, an opportunistic human pathogen, is a commensal species retrieved from the microbiota of almost 30% of humans^10,11^. *S. aureus* colonization is due to host factors and other members of the microbiota that may act as facilitators or antagonists. For example, both *Corynebacterium* spp. and *Staphylococcus epidermidis* have been shown to antagonize nasal colonization by *S. aureus*^12,13^.

A subpopulation of *S. aureus* is able to provoke toxic shock syndrome (TSS), an acute life-threatening illness characterized by sudden onset of fever, cutaneous signs (rash followed by desquamation), hypotension, and multiple organ failure, through the production of TSS toxin-1 (TSST-1). Menstrual TSS (mTSS), a rare but severe disease, is associated with menstruation and tampon use in previously healthy women vaginally colonized by *S. aureus* clones producing TSST-1 (TSST-1^+^)^14–16^. Colonizing *S. aureus* strain use the catamenial products collected in the tampon as a growth medium and proliferate^17^.

Although vaginal colonization by TSST-1^+^ *S. aureus* has been reported in 1% to 4% of the populations studied^18–20^, mTSS afflicts only 1 to 3 in 100,000 women^18,19^. The current assumption of this low prevalence relies on different factors that include the vaginal environment and its resident microbiota. For example, lactobacilli have been shown to be keystone species in vaginal health by strongly affecting the ecology of pathogenic bacteria, controlling their growth and/or virulence^5,21^.

However, most studies have examined variations in the vaginal microbiota during different microbial infections, such as bacterial vaginosis, or even HIV infection^22–25^, but none have examined the influence of vaginal microbial communities on the colonization of *S. aureus* in the vagina and whether an aberrant vaginal microbiota would favour vaginal colonization by *S. aureus*, especially in women using tampons during menses. The oldest studies essentially relied on prokaryote culture, whereas new sequencing techniques are enabling the detection of uncultivable parts of the microbiota. The current challenge is to reconcile both metataxonomic approaches with culturomic analysis in order to assess bacterial interaction theories developed from community analysis (i.e., metabarcoding) by performing experimental bacterial competitions (cultivable microbiome)^26^.

The aim of the present study was to determine the impact of the composition of vaginal microbial communities on tampon colonisation by *S. aureus* during menses. For this purpose, we examined used tampons from healthy women and mTSS cases utilizing culturomic and 16S rRNA gene metabarcoding analyses.

## Methods

### Subjects

Women recruitment, tampons collection and experimental protocols were approved by the ethical committee (Comités de Protection des Personnes (CPP), Interregion Sud-Est, n° L13–159). Ninety-one menstruating healthy volunteers were recruited between March 2014 and August 2016 through the gynaecology departments of Hospices Civil de Lyon. The only inclusion criterion was the use of tampon during menses, regardless of brand. Each volunteer signed informed consent forms. During the same period, 7 mTSS cases in France were spontaneously referred to the National Reference Center for Staphylococci and included in the study. The TSS diagnosis was based on the Centers for Disease Control and Prevention criteria (CDC) diagnostic criteria^27^. In brief, a woman was considered as mTSS if she met all CDC criteria as confirmed or probable TSS: The TSS occurs during menstruation with the use of tampon, the presence of *S. aureus* producing TSST-1 in vaginal specimen, and the absence of focal suppurative *S. aureus* infection and positive blood culture^27^. The clinical and biological characteristics of the cases of mTSS are described in Table S1 in supplementary data.

### Sample collection

A total of 213 tampons were collected from the 91 healthy menstruating women. Forty-eight women provided three tampons (one tampon per menstrual cycle, not necessarily consecutive), 26 provided two tampons, and 17 provided only one tampon. Tampons were placed in a sterile flask and stored at 4 °C until analysis. The conservation at 4 °C never exceed 4 days for analyses in culturomic, beyond this limit the cultivable population decreased dramatically (data not shown). Each tampon was accompanied by an information form about the woman (i.e., age, history of salpingitis or mTSS, antibiotics within the last 15 days, methods of contraception, sexual intercourse within the last 5 days) and tampon (i.e., date of removal, tampon brand, carrying time). For mTSS cases, the incriminated tampon was withdrawn by the doctor or nurse in charge of the patient, placed in a sterile flask, and stored at 4 °C until analysis.

### Menstrual fluid extraction

Menstrual fluid was extracted by suspending the tampon in 15 mL of sterile distilled water and then pressing it. The fluid was aliquoted and maintained as frozen stock (−20 °C) until further analysis. Stocks for the culturomic approach contained 30% glycerol.

### *S. aureus* vaginal screening

*S. aureus* screening was performed by culturing, which has been shown to be more sensitive than PCR^28^, and isolates could be subsequently screened for TSST-1 status. Fifty microliters of menstrual fluid were spread on a SAID chromogenic plate selective for *S. aureus* (chromID™ *S. aureus*, Biomérieux, Marcy l’Étoile, France). Briefly, on this chromogenic medium, *S. aureus* forms distinctive green colonies due to production of α-glucosidase. Other *Staphylococcus* species are developing white colonies or occasionally produce pink colonies due to the hydrolysis of a second chromogenic substrate for β-glucosidase. Real-time SYBR^®^Green biplex PCR *nuc/tst* was performed for each green colony suspected of *S. aureus* for species identification and the co-detection of *S. aureus* nuclease gene (*nuc*) and TSST-1 gene (*tst)*. The primers were as follows: tst1, 5′-GTA AAA GTG TCA GAC CCA CT-3′; tst2, 5′-ATT TTT TTA TCG TAA GCC CTT-3′^29^; nucF, 5′-CAT CCT AAA AAA GGT GTA GAG A-3′; and nucR, 5′-TTC AAT TTT MTT TGC ATT TTC TAC CA-3′^30^. PCR was performed using the LightCycler^®^ Fast Start DNA Master SYBR Green kit (Roche, Meylan, FRANCE) and performed in the LightCycler^®^ thermocycler (Version 4.1) with the following program: denaturation for 30 s at 95 °C, amplification in 35 cycles of 15 s at 95 °C, 20 s at 62 °C, and 30 s at 72 °C, and annealing at 95 °C, 15 s at 65 °C, and 90 °C. The *tst* + *S. aureus* HT20030749 strain belonging to the clone Geraldine was used as a positive control^31^.

For contradictory data on the presence of *S. aureus* in a sample obtained between metabarcoding and culturomic analysis, real presence of *S. aureus* was checked by real-time PCR on metagenomic DNA.

For further analysis, only one tampon per women was included unless *S. aureus* detection differed between tampons (including also differences between *S. aureus tst* + and *S. aureus tst*−carriage). Thus, 108 tampons from healthy women and 7 tampons from mTSS cases were selected to analyse the diversity of the vaginal microbiota during menstruation.

### Culturomic analysis

Culturomic methods consisted of plating menstrual fluids extracted from the 108 tampons on selective media in different atmospheres, followed by colony identification by matrix-associated laser desorption ionization - time of flight (MALDI-TOF) mass spectrometry and 16S rRNA gene sequencing when required. In brief, thawed menstrual fluids, that were less than 4 days old, were plated in diverse media with sterile 10 µL-calibrated spreaders. The media and growth conditions used in this approach are listed in Table 1. Briefly, culture media used are normative chromogenic media that are used in medical diagnosis. Thus, it is known that only a selected range of bacterial species will grow on these specific culture media by forming phenotypically different colony forming units (CFU). These CFU develop morphologies and colours specific to each species or genus on a given selective medium. For each phenotypically different CFU, semi-logarithmic quantification was performed^32^. The logarithmic quantification corresponds to the calibrated loop (10 µL) method that is commonly used to quantify the bacteriuria. The plate reading is in log.mL^−1^ with a limit of detection at 10^2^ CFU.mL^−1^. Identification was performed by MALDI-TOF with the Vitek MS analyser (Biomérieux) and Vitek version 2.3.3 database. When identification failed, 16S rRNA gene (*rrs*) V5/V6/V7 variable region sequencing was performed using the following primers as described by^33^: Neck1, 5′-TCA AAK GAA TTG ACG GGG GC-3′ and Neck2, 5′-GCC CGG GAA CGT ATT CAC-3′. Sequences were analysed by leBIBI^QBPP^ software (Bioinformatics Bacterial Identification - Quick BioInformatic Phylogeny of Prokaryotes, V1.0.1). When identical species was detected in multiple culture mediums, the highest determination was selected to approximate the relative ratio of genera by culturomic analysis. Among the 108 tampons, only 82 could be cultivated, with the other 26 resulting in no growth. The seven mTSS tampons were collected from different French hospitals and processed at the collection site for *S. aureus* isolation and DNA extraction. The culturomic approach was not carried out with these tampons because of the extended delay in transportation to the Centre National de Reference des Staphylocoques.

**Table 1:** Media and growth conditions.

Media	Isolation	Atmosphere	Incubation period *(hours)*
*Candida* ID 2 agar (CAN2, Biomérieux®)	Selective isolation of yeasts and the direct identification of *Candida albicans*	Aerobic	48
Chocolate Agar PolyViteX (PVX, Biomérieux®)	Isolation of fastidious bacteria	5% CO_2_	48
Columbia Blood Agar with 5% Sheep Blood (COS, Biomérieux®)	Selective isolation of fastidious bacteria, detection of haemolysis	Anaerobic	72
Columbia Blood Agar with 5% Sheep Blood, Colistin, and Nalidixic Acid (CNA, Biomérieux®)	Selective isolation of Gram-positive bacteria	5% CO_2_	48
*Gardnerella* Selective Agar with 5% Human Blood (Gadnerella, BD®)	Partially selective isolation of *Gardnerella vaginalis*	5% CO_2_	48
*Lactobacillus* Selection Agar (LBS, BD®)	Partially selective isolation of lactobacilli	Anaerobic	72
Schaedler Kanamycin-Vancomycin Agar with 5% Sheep Blood (Schaedler-KV, BD®)	Selective isolation of fastidious Gram-negative anaerobic microorganisms, especially *Bacteroides* and *Prevotella* species	Anaerobic	72
UriSelect 4 Agar (URI4, BioRad®)	Isolation of urinary pathogens and direct identification of *Escherichia coli*, *Enterococcus* spp., and *Proteus mirabilis*	Aerobic	48

### Metabarcoding analysis

Microbial DNA were efficiently extracted from 107 of the 108 tampons from healthy menstruating women and the 7 tampons from mTSS cases, which were analysed for the diversity of vaginal microbiota by 16S rRNA gene metabarcoding analysis. Briefly, to extract the total microbial DNA from menstrual fluid, 1 mL of the fluid sample was centrifuged at 11,000 × *g* at 4 °C for 10 min. The supernatant was discarded and the pellet suspended in 200 µL of high purity water. Total DNA was extracted using the NucleoSpin Tissue® DNA Isolation Kit (Macherey-Nagel, Hoerdt, France) according to the manufacturer’s instructions. The DNA quality and quantity were assessed by agarose gel electrophoresis and using the Quant-iT™ PicoGreen® dsDNA Assay Kit (Invitrogen, Carlsbad, CA, USA) in 96-well microplates and a TECAN infinite M200 Pro spectrofluorometer (Tecan Trading AG, Männedorf, Switzerland). DNA was adjusted to a concentration of 10 ng/µL and sent to the MR DNA laboratory (Shallowater, TX, USA) for sequencing. The 16S rRNA gene V3/V4 variable region PCR primers 341 F/785 R^34,35^ with a barcode on the forward primer were used in a 28-cycle PCR (5 cycles implemented on PCR products) using the HotStarTaq Plus Master Mix Kit (Qiagen, Valencia, USA) under the following conditions: 94 °C for 3 min, followed by 28 cycles of 94 °C for 30 s, 53 °C for 40 s, and 72 °C for 1 min, with a final elongation step at 72 °C for 5 min. PCR products were checked on a 2% agarose gel to determine amplification success and relative band intensity. Multiple samples were pooled together in equal proportions based on their molecular weight and DNA concentrations. Pooled samples were purified using calibrated Ampure XP beads and used to prepare an Illumina DNA library. Sequencing was performed on a MiSeq following the manufacturer’s guidelines. Sequence data were processed using the MR DNA analysis pipeline. Briefly, sequences were joined, depleted of barcodes, and sequences <150 bp or with ambiguous base calls removed. Sequences were denoised. Operational taxonomic units (OTUs) were defined by clustering at 3% divergence (97% similarity) and chimeras removed. Final OTUs were taxonomically classified using BLASTn against a curated database derived from Greengenes^36^, RDPII (http://rdp.cme.msu.edu), and NCBI (www.ncbi.nlm.nih.gov).

When *S. aureus* was detected in sample only by 16S rRNA gene sequencing, its presence was verified by qPCR targeting *nuc* gene.

### Nucleotide sequence accession numbers

Reads have been deposited in the European Bioinformatics Institute (EBI) database under accession number PRJEB24013.

### Diversity analysis

Sequencing datasets were used to generate rarefaction curves and Shannon, Simpson, and Chao diversity indices. The indices are presented as the average of the individual sample sets (calculated at the OTU level). Extracted from tampons’ fluid, vaginal cultivable bacterial diversity and 16S rRNA gene diversity from the different groups of women (healthy women carrying or not carrying *S. aureus* that produced or did not produce TSST-1) were compared in between-class analysis (BCA)^37^ with the ADE4 and ggplot2 packages for R^38,39^. BCA is a robust alternative to linear discriminant analysis^40^ when the number of samples is too small compared to the number of predictors. When the number of samples is low, and particularly when it is lower than the number of predictors, discriminant analysis cannot be used. In this case, BCA can be very useful and provides illustrative graphical displays of between-group differences^41^. The significance of BCA was assessed using a Monte-Carlo test with 10,000 permutations (null hypothesis: absence of difference between groups). The genera contributing most to differentiating the group of women were identified. Unless otherwise stated, statistical analyses were performed using R version 3.3.2 at *p* < 0.05.

### Comparison of the culturomic and metabarcoding approaches

A co-inertia analysis^42,43^ was performed with the ADE4 software on the 81 samples that were analysed by both culturomic and metabarcoding approaches. Co-inertia analysis comes from Ecological data analysis, and comprises two steps. In the first step, two separate PCA (Principal Component Analysis) are used to find inertia axes and sample scores in each of the two data sets (culturomic analysis and 16S rRNA gene metabarcoding). In a second step, the co-inertia analysis computes new axes that maximize the squared covariance (called “co-inertia”) between PCA sample scores. Co-inertia is a global measure of the relationship between samples of the two data sets: it is high when the two sample scores vary simultaneously (with either a positive or negative relationship), and low when they vary independently. The statistical significance of co-inertia analysis was assessed with a Monte-Carlo test using 10,000 permutations. The null hypothesis of this test was the absence of a relationship between culturomic analysis and 16S rRNA gene metabarcoding scores.

### Ethics approval and consent to participate

Written informed consent was obtained from the volunteers and patients in accordance with the guidelines of the ethical committee (Comités de Protection des Personnes (CPP), Interrégion Sud-Est, France, approval L13–156).

## Results

### Cohort description

The median age of healthy volunteers was 25 years (range 18–52 years). Only one volunteer had a history of salpingitis and two were treated with antibiotics within the 15 days prior to sampling (Table 2). Sixty-nine percent used contraception: 56% (*i.e*. 60 volunteers) the oestroprogestative pill and 13% (*i.e*. 14 volunteers) intrauterine device. Tampax® was the most used tampon brand, followed by Nett® (Table 2). The majority of volunteers (93%; *i.e*. 100 volunteers) did not exceed the recommended maximum use duration of 8 hours. The median delay between tampon collection and analysis was 1 day (range 0–9 days; Table 2).

**Table 2:** Characteristics of the 108 samples used for the diversity analysis of the healthy vaginal microbiota.

Sample	n = 108	
**Age**		
≤25 years	46	42.59%
26–35 years	40	37.04%
>35 years	22	20.37%
**History of salpingitis**	2	1.85%
**History of mTSS**	1	0.93%
**Tampon brand**		
Tampax ®	43	39.81%
Nett®	29	26.85%
Carrefour®	8	7.41%
OB®	5	4.63%
Leader Price®	5	4.63%
Others	18	16.67%
**Tampon use duration**		
*median 4 h 30 [range 27 min-24 h]*		
0–4 h	49	45.37%
4–8 h	51	47.22%
>8 h	7	6.48%
NA	1	0.93%
**Antibiotic use in the previous 15 days**		
Total	2	1.85%
Doxycycline	1	0.93%
Tetralysal	1	0.93%
**Contraceptive method**		
None	33	30.56%
Oestroprogestative pill	60	55.56%
Intra-uterine device	14	12.96%
NA	1	0.93%
**Sexual intercourse in the previous 5 days**		
Yes	40	37.04%
No	66	61.11%
NA	2	1.85%

mTSS, menstrual toxic shock syndrome; NA, not available.

Collected information came from the survey accompanying each sample.

Four of the mTSS cases were confirmed and three probable (Table S1). The median age of mTSS cases was 17 years (range 14–30 years). None of them were treated with antibiotics within 15 days prior to sampling. The mode of contraception was not recorded. Tampax® was the most often used tampon brand.

### *S. aureus* vaginal carriage during menses in women using tampon

The *S. aureus* vaginal carriage status of the 91 volunteers was determined by culturomic analysis from tampons. Sixty-five of the 213 tampons (30.5%) were colonized by *S. aureus* at a given time. Among these, 10 tampons (4.7%) were colonized by a *S. aureus tst* + isolate. But when we relate these values to the 91 volunteers the numbers become 44 volunteers were found to carry *S. aureus* (consistently or transiently; 48% of 91 volunteers), and 6 volunteers (6,6% of 91 volunteers) are carrying an *S. aureus tst* + isolate (consistently or transiently colonized).

Forty-eight volunteers performed follow-up over three menstrual cycles, most generally consecutive. Interestingly, 25 (52% of the 48 volunteers) were colonized by *S. aureus* on at least one of the cycles. Among these 25 carriers, 6 volunteers (24%) had colonization by *S. aureus* over 3 cycles and remaining 19 volunteers (76%) had transient colonization (1 or 2 of the 3 cycles, all combinations were retrieved). When detected, *S. aureus* reached a concentration of 300 to >3 × 10^7^ CFU per tampon (outnumbering the detection technique).

The age of the participants, the tampon brand and duration of use, the delay between tampon collection and analysis, the contraceptive method, and sexual intercourse in the 5 days prior to sampling had no significant effect on *S. aureus* detection (Additional file 1: Table S2). For further analysis, only one tampon per woman was kept, unless a difference in *S. aureus* carriage was found between 2 months. Thus, 108 tampons were selected to analyse the diversity of the vaginal microbiota (Table 2). *S. aureus* strains carrying *tst* were detected in all tampons recovered from mTSS cases and exceeded 3 × 10^7^ CFUs per tampon.

### Culturomic versus metabarcoding to detect *S. aureus* carriage

Sequence analysis of the 16S rRNA gene V3/V4 variable region suggested that *S. aureus* could be retrieved in each samples (Additional file 1: Table S3). However, culturomic analysis determined presence of *S. aureus* only in 44 out of the 91 analysed samples. *nuc* gene (a thermonuclease gene specific to *S. aureus*) real-time PCR performed on the 91 samples confirmed the culturomic data, suggesting that V3/V4 variable region cannot specifically target *S. aureus sensu stricto*. False positive obtained through 16SrRNA gene diversity sequencing data analysis showed from 8 to 78 reads per samples, whereas *S. aureus* positive sample exhibit from 9 reads to 9577 reads. Cultivable analysis also showed to be globally more sensitive than 16S rRNA gene metabarcoding as example the 3 mTSS cases (patient 94, 95 and 96; Additional file 1: Table S3) that showed less than 60 reads (reads similar to false positives) affiliated to *S. aureus* whereas culturomic showed that the bacteria outnumbered the detection limit (>10^7^ CFU/mL).

### Analysis of tampon microbiota diversity during menses by culturomic analysis

Colonies were detected in 82 of the 108 menstrual fluid samples. A total of 51 genera and 96 different species were identified. The number of cultivable bacterial genera colonizing tampons varied from 2 to 11 (median 6), and the number of cultivable bacterial species colonizing tampons varied from 2 to 15 (median 8). The cultivable microbiota composition of 82 tampons is shown in the Additional file 1: Fig. S1B.

**Figure 1:**
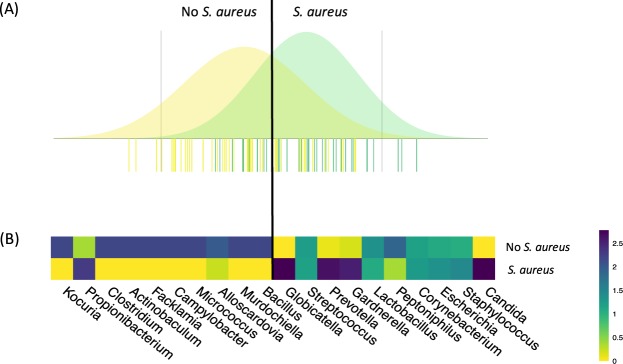
Composition of the vaginal microbiota during menses as determined by culturomic analysis as a function of vaginal colonization by *S. aureus*. (**A**) Between-class analysis was performed at the bacterial genus level. Thirty-six samples were from women vaginally colonized by *S. aureus* (green) and 46 samples from women not colonized by *S. aureus* (yellow). The analysis was supported by a Monte Carlo test (*p* = 0.026). The data include the distribution of bacterial genera according to their contribution to differentiation of the vaginal microbiota of women carrying or not carrying *S. aureus*. (**B**) Heatmap showing the average relative proportion of the 10 microbial genera most commonly associated with vaginal carriage of *S. aureus* (left) and 10 microbial genera most often associated with the absence of *S. aureus* at the vaginal stage (right) in samples containing (lower line) and not containing *S. aureus* (upper line). Heatmap unit is a percentage of the average relative proportion of taxa.

We examined the effect of age, tampon use duration, contraceptive method, and sexual intercourse in the 5 days prior to sampling on the composition of the vaginal cultivable microbiota by BCA. Only tampon use duration exhibited a significant effect on the composition of the vaginal cultivable microbiota (Additional file 1: Fig. S2). Next, the impact of *S. aureus* vaginal carriage on microbiota composition was assessed. BCA of culturomics data indicated that the composition of the vaginal microbiota differed according to the presence of *S. aureus* (Fig. 1A). *Candida*, non*-aureus Staphylococcus, Escherichia, Corynebacterium, Peptoniphilus, Lactobacillus, Gardnerella, Prevotella, Streptococcus*, and *Globicatella* were the bacterial genera most commonly associated with vaginal carriage of *S. aureus*, whereas *Kocuria, Propionibacterium, Clostridium, Actinobaculum, Facklamia, Campylobacter, Micrococcus, Alloscardovia, Murdochiella*, and *Bacillus* were the bacterial genera most often associated with the absence of *S. aureus* (Fig. 1B).

### Analysis of tampon microbiota diversity during menses by metabarcoding analysis

The bacterial diversity of the microbiota was determined by MiSeq Illumina sequencing of the V4 variable region of *rrs*. A total of 261 different bacterial genera were identified from the microbial DNA efficiently extracted from 107 of the 108 tampons from healthy donors and all 7 from mTSS cases. For the healthy donors, an average of 96,251 sequence reads was obtained from each sample (range 42,247–201,056; median 90,622). The number of genera per tampon varied from 29 to 96 (median 59). The proportions of bacterial genera/species found in vaginal microbiota isolated from the different tampons are shown in the Additional file 1: Figs S1A and S3. Clustering of the 107 samples revealed nine different clusters dominated by *Lactobacillus iners* (two clusters), *Shigella sonnei/Escherichia coli*, *Lactobacillus gallinarum* and *Lactobacillus crispatus*, *Gardnerella vaginalis*, *Streptococcus agalactiae* or *Staphylococcus epidermidis*, and a last cluster with vaginal microbiota showing no dominant species (Additional file 1: Fig. S3).

For the seven mTSS donors, an average of 103,808 sequence reads was obtained from each sample (range 45,417–151,011; median 101,049). The number of genera per tampon varied from 50 to 128 (median 72). The proportions of bacterial species found in vaginal microbiota isolated from the different tampons are shown in the Additional file 1: Fig. S3. The bacterial communities of the seven mTSS cases did not cluster together. Three patients had vaginal microbiota clustering with a known cluster. For example, in patient 98 (vaginal mTSS case), the microbiota was in the cluster dominated by *L. iners*, patient 94 in a cluster dominated by *G. vaginalis*, and patient 78 in the cluster dominated by *S. sonnei*/*E. coli*. The four other cases (96, 92, 93, and 97) formed unique branches with multiple bacterial species dominating their vaginal community (Additional file 1: Fig. S3).

Microbiota that were characterized from the tampons of women vaginally colonized with *S. aureus* that did or did not produce TSST-1 and mTSS cases were distributed in many of these groups and did not appear to share the same microbiota composition. Only patient 96, an mTSS case, exhibited a specific community dominated by *Fusobacterium gonidiaformans* and *Prevotella bivia*. The 10 women for whom two tampons were analysed, one colonized by *S. aureus* and the other not, presented vaginal microbiota profiles belonging to different clusters. In three cases, the community changed very little (women 43, 49, and 88), the dominant species remained the same, and the two tampons belonged to the same group. In others, different communities were observed between the two tampons, and the two communities were no longer dominated by the same species. This was the case for woman 28, whose tampon microbiota changed to a community dominated by *L. gallinarum* and *L. crispatus* in the absence of *S. aureus* to a community dominated by *G. vaginalis* when carrying *S. aureus* (Additional file 1: Fig. S3).

**Figure 2:**
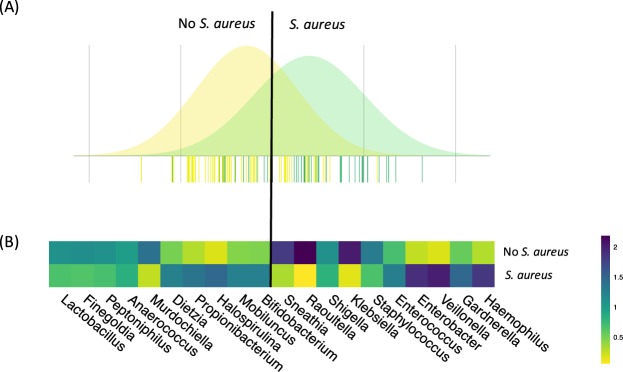
Composition of the vaginal microbiota during menses as determined by 16S rRNA gene metabarcoding analysis as a function of vaginal colonization by *S. aureus*. (**A**) Between-class analysis was performed at the bacterial genus level. Forty-four samples are from women vaginally colonized by *S. aureus* (green) and 63 from women not colonized by *S. aureus* (yellow). The analysis is supported by a Monte Carlo test (*p* = 0.028). Data include the distribution of bacterial OTU according to their contribution to differentiation of the vaginal microbiota of women carrying or not carrying *S. aureus*. (**B**) Heatmap showing the average relative proportion of the 10 bacterial genera most commonly associated with vaginal carriage of *S. aureus* (left) and 10 bacterial genera most often associated with the absence of *S. aureus* at the vaginal stage (right) in samples containing (lower line) and not containing *S. aureus* (upper line). Heatmap unit is a percentage of the average relative proportion of taxa.

As was done for the culturomic analysis, we examined the effect of age, tampon use duration, contraceptive method, and sexual intercourse in the 5 days prior to sampling on the composition of the vaginal microbiota by BCA (Additional file 1: Fig. S2). In the case of the metabarcoding analysis, BCA of 16S rRNA gene sequences indicated that the composition of the vaginal microbiota during menses differed with age (Additional file 1: Fig. S2) and vaginal colonization by *S. aureus* (Fig. 2A). *Haemophilus, Gardnerella, Veillonella, Enterobacter, Enterococcus*, non*-aureus Staphylococcus, Klebsellia, Shigella/Escherichia, Raoultella*, and *Sneathia* were the bacterial genera most commonly associated with vaginal carriage of *S. aureus*, whereas *Lactobacillus, Finegoldia, Peptoniphilus, Anaerococcus*, *Mudochiella, Dietzia, Propionibacterium, Halospirulina, Mobiluncus*, and *Bifidobacterium* were the bacterial genera most often associated with the absence of *S. aureus* at the vaginal stage (Fig. 2B).

The diversity indices calculated at the OTU level are presented in the Additional file 1: Table S4. No significant differences were found based on the presence of *S. aureus* TSST-1 status.

### Correlation of the two methods

**Figure 3:**
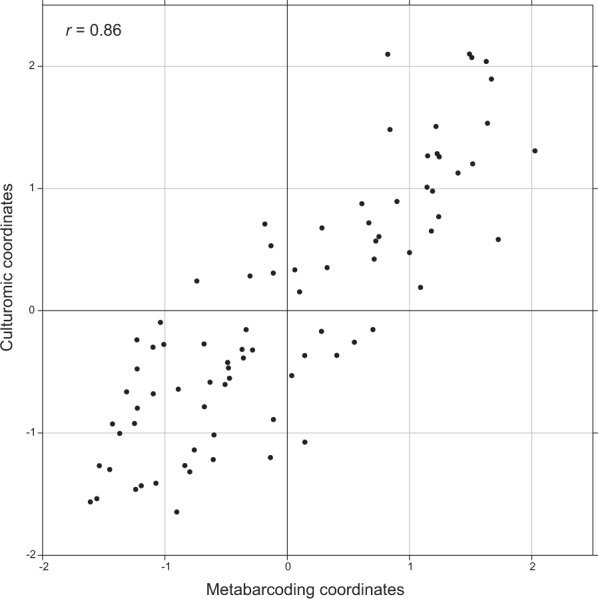
Coinertia analysis between bacterial community data obtained by 16S rRNA metabarcoding (abscissa) and culturomic (ordinate) analyses. Each point on the figure corresponds to one sample (tampon). The value of the correlation coefficient is 0.86. The samples in the upper right-hand corner have high values for the same bacterial species in both metabarcoding and culturomic analyses. Conversely, the samples at the bottom left have low values for the same bacterial species in both metabarcoding and culturomic analyses.

Culturomic and 16S rRNA gene metabarcoding analyses were also evaluated by co-inertia analysis (Fig. 3). The results showed a strong co-structure between the PCA resulting from the culturomic data and the PCA resulting from the metabarcoding data, supported by a significant Monte Carlo test (*p* < 0.05). This result confirms that the bacterial microbiota assessed by culturomic and metabarcoding analyses are closely linked.

As the data from the BCAs analysis showed some common taxa between culturomic and metabarcoding, the microbiota fractions from the 81 samples tested by both culturomic and 16S rRNA gene metabarcoding analysis were compared (Additional file 1: Fig. S1). The relative proportion of each common genus between the two methods is represented for each sample and the ratio calculated for each genus, excluding other. When considering the common genera between both techniques, the mean ratios were not significantly different from 1 (Student’s test), indicating a correspondence between the two methods (Additional file 1: Fig. S1C).

## Discussion

The vaginal bacterial community in apparently healthy reproductive age women has been shown to present a strong barrier against various infections^5,44–46^, but its role in preventing *S. aureus* colonization or virulence is poorly understood. mTSS is a rare but specific disease caused by TSST-1-producing *S. aureus* in women who use tampons or a menstrual cup as periodic protection^15,47,48^. Colonizing *S. aureus* strain use the catamenial products collected into the intra vaginal device as a growth medium and proliferate^17,49,50^.

The aim of the present study was to determine whether the composition of microbial communities that colonised tampon during menstruation is associated to the vaginal carriage of *S. aureus* and TSST-1-producing *S. aureus*.

mTSS is more prevalent in young women. We initially though that age, though hormonal changes, has an impact on vaginal microbiological communities that interfere with *S. aureus* carriage. Our results do not support this hypothesis and that the high prevalence of mTSS in young women population is linked to higher carriage of *S. aureus* in this population^15^.

*S. aureus* carriage was assessed by cultivable methods, which have been described to be more sensitive than PCR techniques^28^ and allowed subsequent screening of the presence of *tst*. Thus, we detected tampon colonization by *S. aureus* in 40.7% of tampons from healthy women, which is higher than the previous determination of 9–28% in menstruating women based on vaginal sampling by swab instead of tampons^51,52^. None of the clinical data recorded in our study appeared to affect vaginal *S. aureus* carriage in healthy women (i.e., age, tampon brand, duration of use, contraceptive method, sexual activity in the 5 days prior to sampling). Among the carriers of *S. aureus*, 24% were positive during three consecutive menstruations. This result is startlingly similar to that observed for *S. aureus* nasal colonization with approximately 30% of permanent *S. aureus* carriers and 70% intermittent carriers^10,11^.

We determined that TSST-1^+^ *S. aureus* colonized 5.7% of the tested tampons, which is a little higher but comparable to previous studies reporting vaginal colonization by TSST-1^+^ *S. aureus* in 1% to 4% of the woman studied^18–20^. Interestingly, although *S. aureus* was found by culture (from 300 CFU/mL to >10^5^ CFU/mL), it could not always be correctly retrieved by the 16S RNA gene sequencing approach, suggesting that the bacteria did not reach the detection level. Even in tampons from mTSS cases, in which culture approaches estimated colonization of >10^7^ CFU/mL, the bacterium was detected below 60 sequences by meta-analysis for patients 94, 95, and 96 (Additional file 1: Fig. S3). These data were consistent with previous studies showing that, in complex ecosystems such as the gut microbiota, culture-independent techniques are unable to detect bacteria <10^6^ cells/mL^28^. The present data highlight that TSST-1^+^ *S. aureus* is not necessarily the most abundant taxa in the vaginal of mTSS cases.

Recent community profiling by metataxonomic sequencing techniques have shown that healthy vaginal microbial communities are usually dominated by *Lactobacillus* (*L. crispatus, L. iners, L. gasseri, L. jensenii*) or mixed profiles depleted of lactobacilli and consisting of bacteria such as *Prevotella, Gardnerella, Atopobium, Megasphaera*, and *Streptococcus*^24,53–57^. The results of our 16S RNA gene study demonstrated that besides the currently published community states type (CST; vaginal microbiota which are dominated by one or more species  50% of all sequences in a sample^24,55,56^), new state dominated by different populations exist on healthy women’s tampons. *L. gasseri* (CST II) and *L. jensenii* (CST V), which are prominent in other studies^24,35^, were not dominant organisms in any microbiota profile in the current study. Only *L. crispatus* (CST I), *L. iners* (CST III) and *G. vaginalis* (CST IV) dominating communities were already described in previous studies^24,55,56^. But new CST dominated by *Escherichia*/*Shigella*, *Streptococcus agalactiae* CST or *S. epidermis* were characterized (Fig. S3). These divergences from previous studies may rely on different factors, such as socio-demographics, sexual activity, health behaviour, and hygiene, but the most important factor that distinguishes the current study from previous studies was the sampling technique. To the best of our knowledge, previous studies all relied on swab sampling of the vaginal microbiota, whereas we directly studied the tampon colonization. Tampons were also in contact with skin hand microbiota when they were inserted and removed without any other protection than hand washing. Besides, tampons constitute a complex environment for bacteria. They are not uniformly impregnated by menstrual fluid, creating micro-niches enabling the development of specific metabolism and interactions, such as the production of TSST-1^14^.

However, consistent with previous studies assessing vaginal microbial diversity, we found that the tampon microbiota varied largely among healthy women and were, in a certain manner, specific to each woman^24,35,54–56,58^. Most women (11/16) with differential carriage of *S. aureus* between two menses presented changes in the microbiota composition. Tampons from two different cycles had a conserved community dominated by the same species in only five women. This corroborated previous studies showing that the vaginal microbiota may shifted to different community state through menstrual cycles^35,55,58^. In contrast to our expectations, clustering analysis did not distinguish a specific cluster grouping a microbial community carrying TSST-1^+^ *S. aureus* or the microbiota of mTSS cases. In addition, the richness, diversity, and ecological distance were similar between *S. aureus* carriers and non-carriers. These results suggest that neither variation in community composition nor constantly high levels of apparent diversity are indicative of *S. aureus* carriage status. Thus, the tampon microbiota composition that may control the ecology of TSST-1^+^ *S. aureus* and its virulence during toxic shock syndrome either directly by controlling (positively or negatively) its growth and/or TSST-1 production^21^, or indirectly by modifying the vaginal environmental conditions (i.e., pH, carbon, O_2_ levels)^59^, is not completely reshaped when *S. aureus* is present.

Carriage of *S. aureus* has a greater impact on the non-dominant population in the microbiota. BCA of the microbiota defined by the culturomic approach and *rrs* metabarcoding analysis showed that the microbiota of healthy menstruating women differed according to their *S. aureus* carriage status (Figs 1 and 2). Several bacterial genera, such as *Murdochiella* and *Enterobacter*, were preferentially found in the absence of *S. aureus* carriage, whereas non-aureus *Staphylococcus* and *Bifidobacterium* were more abundant in the presence of *S. aureus* carriage. Notably, *Lactobacillus*, a genus often described as an inhibitor of the growth of several pathogens, including *S. aureus*^58,59,60,61^, was not specifically associated with the carriage status. Here, we found that, even in the microbiota of tampons from mTSS cases, lactobacilli could be predominant. This observation contradicts some studies suggesting that the vaginal microbiota of healthy women is predominantly colonized by lactobacilli and protect against mTSS, whereas in the case of dysbiosis, such as vaginosis, populations of lactobacilli are overgrown by other bacterial populations^21,62^. Unlike lactobacilli, *S. agalactiae* significantly co-presents with *S. aureus* in pregnant women^63^ and has been shown to induce toxin production in *S. aureus*^21^. Although *S. agalactiae* was co-identified with *S. aureus* in the current study, it was not abundant in mTSS cases, suggesting that abundance of this bacterium does not correlate with an increased risk of developing mTSS.

These observations call into question whether tampon microbiota profiling studies may be reliably used to link vaginal bacterial community composition to health outcomes. Taxonomic composition is not sufficient to decipher the functional outcomes of a given bacterial community. A possible way of studying microbiota functioning and interactions is to return to *in vitro* studies combining larger sets of bacterial populations. To date, the vaginal microbiota has been shaped predominantly by culture-based or 16S rRNA gene diversity sequencing studies. Although both approaches have some limitations, they contribute to a wealth of information on microbiota composition. 16S rRNA gene-based studies are known to gain access to a large diversity of uncultivable bacteria, but have amplification biases and, for some, taxa-limited resolution^63^. Culturomic analysis associating bacteriological culture with characterization by MALDI-TOF enables the identification of large sets of bacteria populations^28,64–67^, but is heavy to handle since large sets of culture media and culture conditions have to be tested and give a approximation of target the cultivable fractions (approximation for enumeration). Here, we analysed the bacterial community by *rrs* gene sequencing and a culturomic approach for the menstrual fluids extracted from 81 tampons. We demonstrated that both techniques provided data that significantly correlated and their analyses reached the same conclusions. Thus, in future studies, it would be interesting to obtain a set of cultivable bacteria to pursue competition assays combining culture and sequencing approaches.

In conclusion, we showed that shifts in microbiota composition consist not only of changes in the dominance of lactic acid-producing bacterial species^55^, but also in metabarcoding-undetectable species, such as TSST-1^+^ *S. aureus*, which could greatly impact the health of the patient.

## Electronic supplementary material

Supplementary information

Datasets
