# Author Correction: Complex ecological interactions of *Staphylococcus aureus* in tampons during menstruation

**DOI:** 10.1038/s41598-020-57947-2

**Published:** 2020-01-30

**Authors:** Isaline Jacquemond, Anaëlle Muggeo, Gery Lamblin, Anne Tristan, Yves Gillet, Pierre Adrien Bolze, Michèle Bes, Claude Alexandre Gustave, Jean-Philippe Rasigade, François Golfier, Tristan Ferry, Audrey Dubost, Danis Abrouk, Samuel Barreto, Claire Prigent-Combaret, Jean Thioulouse, Gérard Lina, Daniel Muller

**Affiliations:** 1 Université de Lyon, Université Claude Bernard Lyon 1, CNRS, INRA, VetAgro Sup, UMR Ecologie Microbienne, 43 bd du 11 Novembre, F-69622 Villeurbanne, France; 2 CIRI, Centre International de Recherche en Infectiologie, Inserm U1111, Université Lyon 1, Ecole Normale Supérieure de Lyon, CNRS UMR 5308, Lyon, France; 3 Department of Gynecology, Hospices Civils de Lyon, Hôpital Femme Mère Enfant, Bron, France; 4 Centre National de Référence des Staphylocoques, Institut des Agent infectieux, Hôpital de la Croix Rousse, Hospices Civils de Lyon, Lyon, France; 5 Department of Pediatric Emergency, Hospices Civils de Lyon, Hôpital Femme Mère Enfant, Bron, France; 6 Department of Gynecological Surgery and Oncology, Obstetrics, Hospices Civils de Lyon, Centre Hospitalier Lyon Sud, Pierre Bénite, France; 7 Service des maladies infectieuses et tropicales, Hôpital de la Croix Rousse, Hospices Civils de Lyon, Lyon, France; 8 Université Lyon 1, CNRS, UMR5558, Laboratoire de Biométrie et Biologie Evolutive, Villeurbanne, France

Correction to: *Scientific Reports* 10.1038/s41598-018-28116-3, published online 02 July 2018

In the Supplementary Information file originally published with this Article, the Supplementary Figures S1, S2 and S3 were omitted. These errors have been corrected in the Supplementary Information that now accompanies the Article.

