# Supplementary material for: Complex ecological interactions of Staphylococcus aureus in tampons during menstruation
Source: Sci Rep. 2018 Jul 2;8:9942. doi: 10.1038/s41598-018-28116-3 (PMC6028614; doi:10.1038/s41598-018-28116-3)
Supplement: Supplementary file 1 — Supplementary information [file 41598_2018_28116_MOESM1_ESM.pdf]

Complex ecological interactions of *Staphylococcus aureus* in tampons during menstruation.

Isaline Jacquemond[1,2], Anaëlle Muggeo[2], Gery Lamblin[3], Anne Tristan[2,4], Yves Gillet[2,4,5], Pierre Adrien Bolze[6], Michèle Bes[4], Claude Alexandre Gustave[2,4], Jean-Philippe Rasigade[2,4], François Golfier[6], Tristan Ferry[7], Audrey Dubost[1], Danis Abrouk[1], Samuel Barreto[1,8], Claire Prigent Combaret[1], Jean Thioulouse[8], Gérard Lina[2,4*], Daniel Muller[1*]

Table S1. Clinical and biological characteristics of the cases of menstrual toxic shock syndrome included in the study.

Patient	Case 1	Case 2	Case 3	Case 4	Case 5	Case 6	Case 7
**Sample nb**	92	93	94	95	96	97	98
**Age (year)**	18	17	30	21	14	15	17
**Tampon brand**	Tampax	NR	Tampax	NR	NR	Tampax	Net
**Time of carriage**	7h	Overnight	Overnight	24h	4h	Overnight	Overnight
**Hospitalisation unit**	ICU	ICU	ICU	ICU	ICU	Medicine	ICU
**TSS CDC criteria**	Probable	Probable	Probable	Probable	Confirmed	Probable	Probable
**Fever**	Yes	Yes	Yes	Yes	Yes	Yes	Yes
**Hypotension**	Yes	Yes	Yes	Yes	Yes	Yes	Yes
**Cutaneous rash**	Yes	Yes	Yes	Yes	Yes	Yes	Yes
**Desquamation**	NR	NR	NR	NR	Yes	NR	Yes
**Number of system involvement**	3/7	3/7	6/7	4/7	3/7	5/7	4/7
***S. aureus* strain ref.**	ST20141074	ST20140803	ST20160181	ST20151989	ST20151978	ST20160223	ST20161423
***S. aureus* genotype**	CC5 *tst+*	CC30 *tst+*	CC30 *tst+*	CC30 *tst+*	CC30 *tst+*	CC30 *tst+*	CC22 *tst+*

ICU, Intensive Care Unite; NR, Not Recorded. *S. aureus* was genotyped using diagnostic DNA microarrays and identibac *S. aureus* Genotyping® (Alere) [2].

Table S2. Demographic and clinical data.

Factor		*S. aureus* vaginal carriage	
		Yes	No
**Age** ***(years)***			
	≤ 25	21	25
	26-35	14	26
	> 35	9	13
**Tampon brand**			
	Tampax®	19	24
	Nett®	10	19
	Other	15	21
**Tampon use duration** ***(hours)***			
	0-4	19	30
	4-8	19	32
	> 8	5	2
**Sexual intercourse in the previous 5 days**			
	Yes	16	24
	No	27	39
**Contraceptive method**			
	OP pill	23	37
	IUD	7	7
	None	13	20
**Delay between tampon collection and analysis** ***(days)***			
	0	5	26
	1	17	27
	2	9	7
	3	3	3
	4	3	1
	5	1	0
	6	2	0
	7	1	0
	8	1	0
	9	1	0

OP, oestroprogestative; IUD, intra-uterine device.

Table S3. Comparison of 16S rRNA gene sequencing approaches to the culturomic approach for quantifying *Staphylococcus aureus* abundances

Sample number	Menstrual toxic shock[a]	*nuc* PCR[b]	*tst* PCR[c]	*S. aureus* abundance	
				Metabarcoding data (reads)	Culturomic data (CFU/mL)
**92**	1	1	1	9527	10000000
**93**	1	1	1	5327	10000000
**94**	1	1	1	9	10000000
**95**	1	1	1	140	10000000
**96**	1	1	1	60	10000000
**97**	1	1	1	1331	10000000
**7a**	0	1	1	28	< 100
**11**	0	1	1	1719	10000000
**24**	0	1	1	17	1000
**52**	0	1	1	93	10000000
**1a**	0	1	0	55	100000
**10**	0	1	0	15	10000
**16**	0	1	0	5070	10000000
**19a**	0	1	0	1240	10000000
**22**	0	1	0	138	1000000
**23a**	0	1	0	8	< 100
**28a**	0	1	0	14	< 100
**30a**	0	1	0	13	100
**33**	0	1	0	178	100000
**36**	0	1	0	9	10000
**37**	0	1	0	6	< 100
**38**	0	1	0	149	1000000
**39**	0	1	0	180	100000
**42**	0	1	0	6	1000000
**43a**	0	1	0	36	1000
**44a**	0	1	0	47	< 100
**45**	0	1	0	131	100000
**46**	0	1	0	19	< 100
**48**	0	1	0	75	10000000
**49a**	0	1	0	53	1000
**51**	0	1	0	654	10000
**53a**	0	1	0	81	100
**59a**	0	1	0	35	1000000

**71a**	0	1	0	7	100
**75**	0	1	0	6	< 100
**77**	0	1	0	46	100
**79a**	0	1	0	6	100
**82**	0	1	0	441	10000000
**83a**	0	1	0	21	100000
**85**	0	1	0	21	100000
**88a**	0	1	0	17	100
**89a**	0	1	0	37	10000000
**1b**	0	0	0	8	0
**2**	0	0	0	4	0
**3**	0	0	0	6	0
**4**	0	0	0	14	0
**5**	0	0	0	18	0
**6**	0	0	0	7	0
**7b**	0	0	0	26	0
**8**	0	0	0	57	0
**13**	0	0	0	60	0
**14**	0	0	0	14	0
**17**	0	0	0	59	0
**18**	0	0	0	6	0
**19b**	0	0	0	11	0
**20**	0	0	0	39	0
**23b**	0	0	0	7	0
**25**	0	0	0	11	0
**26**	0	0	0	15	0
**28b**	0	0	0	6	0
**32**	0	0	0	23	0
**34**	0	0	0	47	0
**41**	0	0	0	8	0
**43b**	0	0	0	37	0
**44b**	0	0	0	26	0
**59b**	0	0	0	2	0
**64**	0	0	0	41	0
**65**	0	0	0	22	0
**66**	0	0	0	13	0
**67**	0	0	0	2	0
**68**	0	0	0	5	0
**69**	0	0	0	16	0
**70**	0	0	0	13	0

**71b**	0	0	0	38	0
**72**	0	0	0	6	0
**73**	0	0	0	46	0
**74**	0	0	0	8	0
**76**	0	0	0	15	0
**78**	0	0	0	38	0
**79b**	0	0	0	6	0
**80**	0	0	0	11	0
**81**	0	0	0	7	0
**83b**	0	0	0	22	0
**84**	0	0	0	26	0
**87**	0	0	0	13	0
**88b**	0	0	0	5	0
**89b**	0	0	0	78	0

[a] 1= Menstrual toxic shock ; 0 healthy woman.
[b]*nuc* PCR targets thermonuclease gene specific to *S. aureus* (1= positive; 0 =negative).
[c] *tst* PCR target the gene encoding TSST-1 (1= positive; 0 =negative).

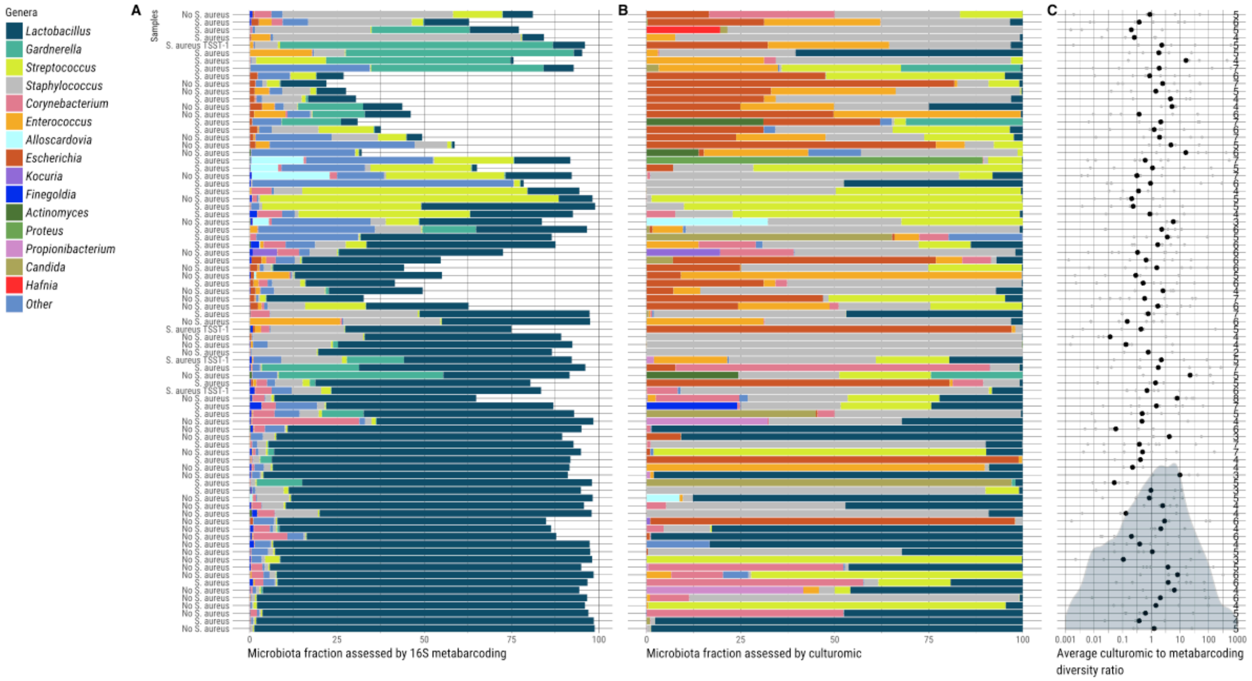

Figure S1. Analysis of the bacterial genera colonizing tampons in healthy women during menstruation. (A) 16S rRNA gene sequences analyses. (B) Culturomic analyses. Each line corresponds to a tampon (n = 81). Tampons were clustered according to their genera composition in (A), and the same order retained in (B) and (C). For each sample, the carriage status of *S. aureus* is indicated (No *S. aureus*, *S. aureus,* or *S. aureus* TSST-1). The 15 most abundant and common genera between the two methods are represented. To simplify the figure, the Other group defines the set of taxa common to both techniques but were not part of the 15 most abundant. In (A), remaining white indicates the percentage of taxa that are not retrieved in both methods. The relative proportion of each genus is represented for each sample analysed by metabarcoding (PM) and culturomic (PC) and the PM/PC ratio calculated ("Other" excluded). Ratios close to 1 exhibited good correspondence between both methods. (C) The PM/PC ratios for each common taxa is represented as grey dot and the average PM/PC ratio as black dot. The average of all finites and non-null ratios were not significantly different from 1 (Student's test, p=0.21). Numbers shown are number of common taxa used to calculate the average PM/PC ratio.

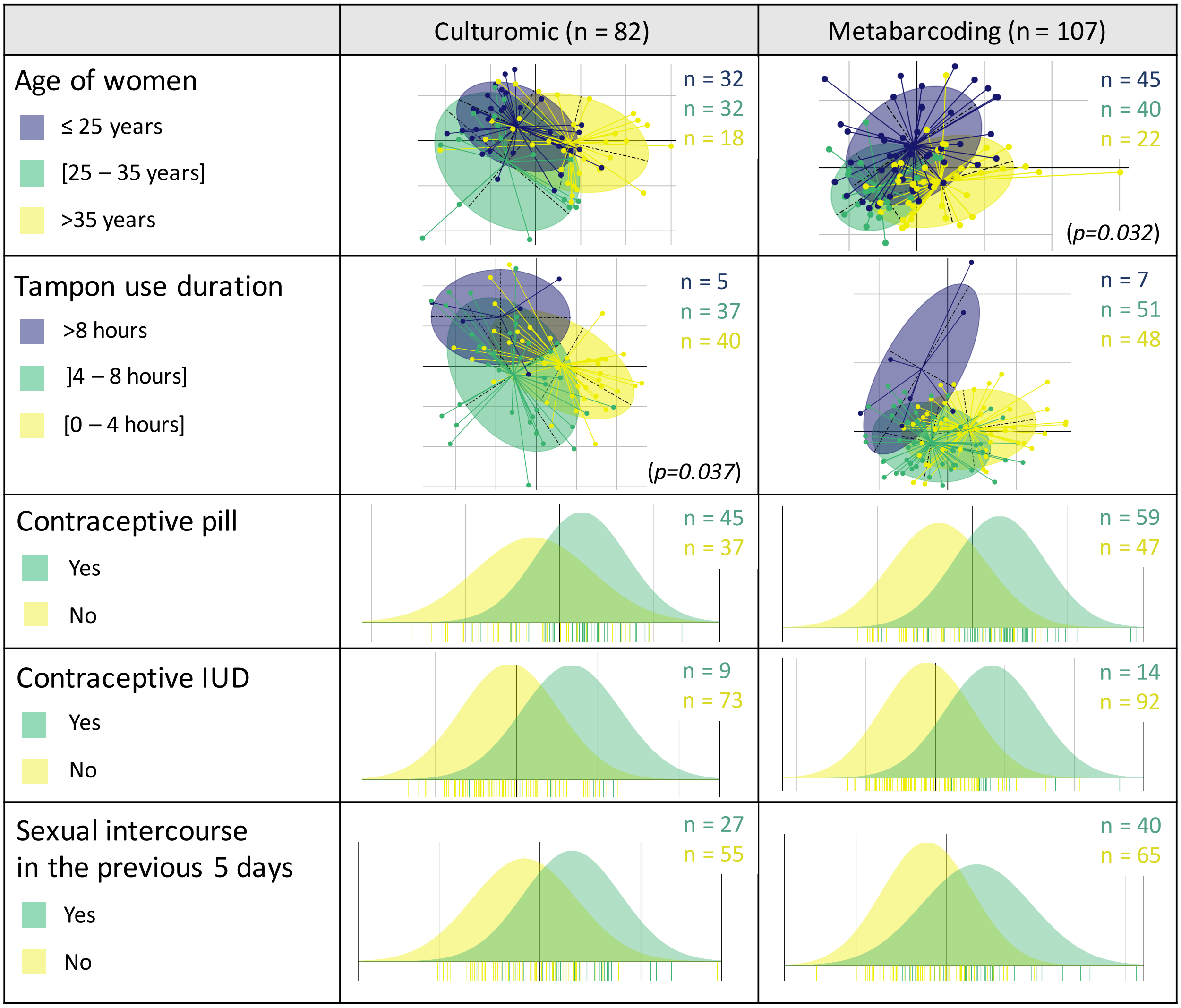

Figure S2. Effect of various factors on the diversity of the vaginal microbiota of healthy women during menstruation analysed by metabarcoding and culturomic approaches. Between-class analysis was performed at the genus level. Colours distinguish between samples belonging to factor groups (age class, carrying time, contraception). The more separation between the colour groups, the more the tested factor impacts the composition of the community. Statistical differences (when any) are indicated (Monte-Carlo test ; $p < 0.05$).

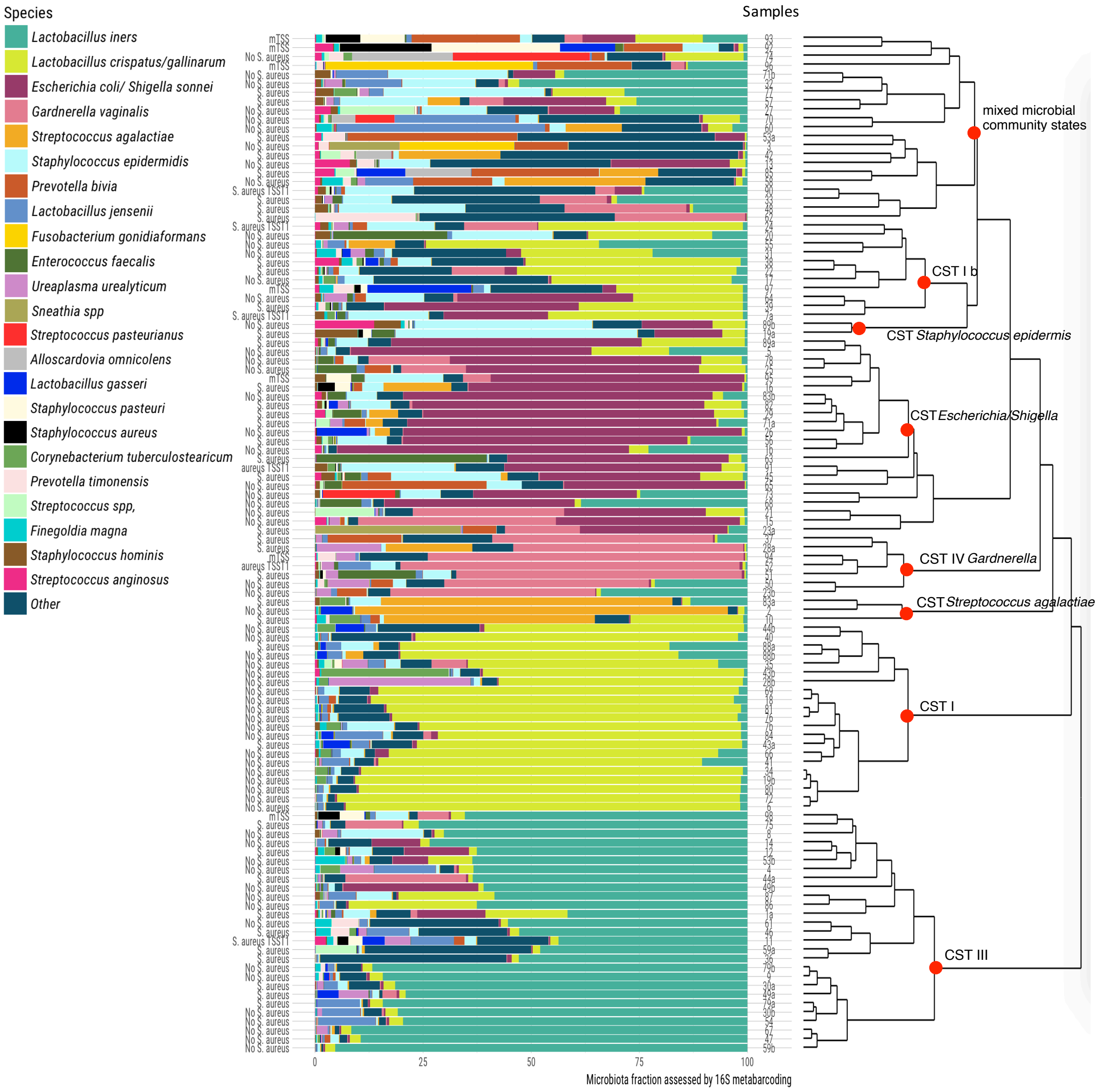

Species
Lactobacillus iners
Lactobacillus crispatus/gallinarum
Escherichia coli/ Shigella sonnei
Gardnerella vaginalis
Streptococcus agalactiae
Staphylococcus epidermidis
Prevotella bivia
Lactobacillus jensenii
Fusobacterium gonidiaformans
Enterococcus faecalis
Ureaplasma urealyticum
Sneathia spp
Streptococcus pasteurianus
Alloscardovia omnicolens
Lactobacillus gasseri
Staphylococcus pasteuri
Staphylococcus aureus
Corynebacterium tuberculostearicum
Prevotella timonensis
Streptococcus spp,
Finegoldia magna
Staphylococcus hominis
Streptococcus anginosus
Other
Samples
0
25
50
75
100
Microbiota fraction assessed by 16S metabarcoding
mixed microbial community states
CST I b
CST Staphylococcus epidermis
CST Escherichia/Shigella
CST IV Gardnerella
CST Streptococcus agalactiae
CST I
CST III

Figure S3. Analysis of bacterial species colonizing tampons in healthy women and mTSS cases during menstruation by *rrs* gene sequence analyses. Each line represents a tampon and its relative composition in bacterial species. For each sample, the carriage status of *S. aureus* is indicated (no *S. aureus*, *S. aureus*, *S. aureus tst+*), and mTSS specifically indicates mTSS cases carrying S. aureus TSST-1. Some tampons came from the same woman and are indicated by the same sample number (see column samples), "a" meaning that the tampon was colonized by S. aureus and "b" meaning that it was not. The tree was generated using the complete link algorithm and the Euclidean distances between the different tampons' microbial communities. The Other group defines the set of taxa that were not part of the 23 most abundant. Community state type (CST) shown based either on previously literature (Ravel et al 2011) CSTI and CSTIb are dominated by *L. crispatus*, CSTIII by *L. iners*, CSTIV by *G. vaginalis*, or as new CTS fellowed by the community dominating OTUs' affiliation name.
